# Are Serum 25-Hydroxyvitamin D Deficiency and Insufficiency Risk Factors for the Incidence of Dynapenia?

**DOI:** 10.1007/s00223-022-01021-8

**Published:** 2022-09-15

**Authors:** Maicon Luís Bicigo Delinocente, Mariane Marques Luiz, Dayane Capra de Oliveira, Aline Fernanda de Souza, Paula Camila Ramírez, Roberta de Oliveira Máximo, Natália Cochar Soares, Andrew Steptoe, Cesar de Oliveira, Tiago da Silva Alexandre

**Affiliations:** 1 Post Graduate Program in Gerontology, Federal University of São Carlos, São Carlos, Brazil; 2 Post Graduate Program in Physiotherapy, Federal University of São Carlos, São Carlos, Brazil; 3 Escuela de Fisioterapia, Universidad Industrial de Santander, Bucaramanga, Colombia; 4 Department of Epidemiology and Public Health, University College London, London, UK; 5 Department of Gerontology, Federal University of São Carlos, Rod. Washington Luís, km 235, São Carlos, São Paulo, SP-310 Brazil

**Keywords:** 25-Hydroxyvitamin D, Incidence, Dynapenia, Musculoskeletal aging

## Abstract

Epidemiological evidence showing the association between low 25(OH)D and age-related reduction in neuromuscular strength (dynapenia) is a paucity and controversial and, to date, the effect of osteoporosis and vitamin D supplementation on these associations has not been measured. Thus, we analyze whether serum 25(OH)D deficiency and insufficiency are risk factors for the incidence of dynapenia in individuals aged 50 or older and whether osteoporosis or vitamin D supplementation modify these associations. For that, 3205 participants of the ELSA study who were non-dynapenic at baseline were followed for 4 years. Vitamin D was measured at baseline by the serum concentration of 25(OH)D and classified as sufficient (> 50 nmol/L), insufficient (≥ 30 and ≤ 50 nmol/L) or deficient (< 30 nmol/L). The incidence of dynapenia was determined by a grip strength < 26 kg for men and < 16 kg for women at the end of the 4-year follow-up. Poisson regression models were adjusted by sociodemographic, behavioral, clinical and biochemical characteristics. Serum 25(OH)D deficient was a risk factor for the incidence of dynapenia (IRR = 1.70; 95% CI 1.04–2.79). When only individuals without osteoporosis and those who did not use vitamin D supplementation were analyzed, both serum 25(OH)D deficiency (IRR = 1.78; 95% CI 1.01–3.13) and insufficiency (IRR = 1.77; 95% CI 1.06–2.94) were risk factors for the incidence of dynapenia. In conclusion, a serum level of 25(OH)D < 30 nmol/L is a risk factor for the incidence of dynapenia. Among individuals without osteoporosis and those who do not take vitamin D supplementation, the threshold of risk is higher (≤ 50 nmol/L).

## Introduction

Independent of ethnicity and geographic location, more than half the world’s population has vitamin D insufficiency or deficiency, which is measured by serum levels of 25-hydroxyvitamin D [25(OH)D] [1, 2]. This condition is even more prevalent in older adults due to the greater occurrence of chronic diseases, use of medicine, presence of functional limitations, less exposure to sunlight and impairment in the production of 7-dehydrocholesterol in this group [2, 3].

There is evidence that bone and muscle tissues are interconnected not only mechanically and physically [4–8], but also biochemically via paracrine and endocrine communication [5–7]. Thus, endocrine disorders, such as serum 25(OH)D insufficiency and deficiency could provoke an imbalance in protein synthesis that would culminate in the loss of bone mineral density as well as reductions in muscle mass, strength (dynapenia) and function [3, 4, 6, 9].

Dynapenia is an important risk factor for disability and mortality later in life [9, 10] that is partially explained by muscle atrophy [10]. This condition has recently been associated with low serum 25(OH)D concentrations in cross-sectional studies [11–15] and is justified because serum 25(OH)D deficiency may culminate in a reduction in Ca^2+^ uptake in myocytes, exerting a negative impact on muscle contraction kinetics and leading to failures in myogenesis and the expression of genes responsible for the growth, metabolism and differentiation of myoblasts, which hinders the repair process and metabolism of muscle fibers [16, 17].

In longitudinal studies, this association has not been confirmed [15]. Bislev Grove-Laugesen and Lars Rejnmark demonstrated, in a meta-analysis, that vitamin D supplementation would not be able to promote muscle health, as measured by handgrip strength. However, most of the studies evaluated had many biases such as the small number of studies that included men, had important methodological and analysis differences, as well as there were many differences in the dosage, treatment time and form of administration of vitamin supplementation D. Moreover, the majority (83%) of the participants had serum 25(OH)D sufficiency (>50 nmol/l) at baseline, which greatly limits the conclusions found. Finally, subgroup analyzes of the meta-analysis suggest that vitamin D supplementation may be beneficial only in individuals with low serum 25(OH)D levels [18].

Therefore, guided by these inconsistencies of the studies to date, two hypotheses were tested in the present study: (a) serum 25(OH)D deficiency is a risk factor for the incidence of dynapenia in individuals aged 50 or older in a 4-year follow-up period; (b) as a portion of the population older than 50 years of age has osteoporosis and, therefore, takes vitamin D supplementation, the exclusion of this group of individuals would demonstrate that the threshold of the association between low serum 25(OH)D levels and the risk of dynapenia is even higher.

## Methods

### Study Population

The data analyzed in the present investigation were from the English Longitudinal Study of Ageing (ELSA), which is a panel study with a representative sample of the population aged 50 years or older residing in England. The study began in 2002 with a sample composed of participants from the Health Survey for England. Interviews were conducted with the use of questionnaires every 2 years. Biochemical exams and physical performance measures were collected by a nurse and repeated every 4 years. More details on the ELSA study can be found in previous publications [19, 20].

The sample of the present investigation was composed of 3205 non-dynapenic individuals aged 50 or older who participated in the ELSA study in 2012 (wave 6), which was the first year that serum 25(OH)D levels were collected. All participants signed a statement of informed consent. ELSA received approval from the National Research Ethics Service—London Multicenter Research Ethics Committee.

### Serum Concentrations of 25-Hydroxyvitamin D [25(OH)D]

The serum concentration of 25(OH)D was collected for the first time in ELSA in 2012, which was considered baseline for the present investigation. Blood samples were collected by nurses and the determination was performed by chemiluminescence (Diasorin Liaison Immunoassay) at the Royal Victoria Infirmary (Newcastle upon Tyne, United Kingdom), which participates in the Vitamin D External Quality Assessment Scheme. The serum 25(OH)D assay has an analytical sensitivity of 7.5 nmol/L, with a coefficient of variation from 8.7 to 9.4%. All assays were performed in duplicate. In the present analyses, serum 25(OH)D concentrations were categorized based on the US Institute of Medicine (IOM): sufficient (> 50nmol/L), insufficient (≥ 30 and ≤ 50nmol/L) and deficient (< 30nmol/L) [21, 22].

### Neuromuscular Strength—Dynapenia

Grip strength was measured using a handheld dynamometer (*Smedley’s for Hand*) with a scale from 0 to 100kg. The dynamometer was adjusted to the size of each participant’s hand. Three trials were performed with a one-minute interval between trials. The highest value achieved on the dominant hand was considered in the analyses [19, 20, 23].

Only individuals without dynapenia (grip strength ≥ 26kg for men and ≥ 16kg for women) at baseline were included in the present investigation. Individuals with grip strength < 26kg for men and < 16kg for women after 4 years of follow-up were considered incident cases of dynapenia [23].

### Covariates

The covariates collected at baseline were selected based on associations between the reduction in neuromuscular strength and serum 25(OH)D concentrations reported in previous studies [21–27].

The sociodemographic characteristics considered were sex, age (50–59; 60–69; 70–79; 80 years or more), educational level (0–11; 12–13; > 13 years), quintiles of total wealth (sum individual’s or a household’s total financial assets and net worth [assets—debts] accumulated over their lifetime), being classified from the highest (1st quintile) to the lowest wealth (5th quintile), ethnicity (white or non-white) and condition of living or not living alone [26, 27].

Health behavior variables included were physical activity level, smoking status and alcohol intake. Physical activity level was evaluated using three questions taken from a validated instrument used in the *Health Survey for England*. The participants were asked about the frequency with which they practiced exercise (once per week, more than once per week; one to three times were more or hardly ever/never) and the intensity of exercise. Activities were classified as vigorous (running, swimming, cycling, tennis, aerobics, weightlifting), moderate (gardening, washing the car, walking at a moderate pace, dancing, stretching) and light (vacuuming the home, washing clothes, household repairs). The individuals were classified as physically inactive/having a sedentary lifestyle (no weekly activity) or active (light, moderate or vigorous activity at least once per week) [26]. With regards to their smoking status, individuals were classified as non-smokers, ex-smokers or smokers. In terms of weekly alcohol intake, participants were classified as those who drank alcoholic beverages up to once a week, those who drank two to six times a week, and those who drank daily [26].

Health conditions were evaluated based on self-reports of having a medical diagnosis of stroke, osteoarthritis, cancer, heart disease, lung disease and osteoporosis [26, 27]. Diabetes and systemic arterial hypertension were defined based on self-reports of a medical diagnosis. These reported conditions were confirmed by the medications taken and glycated hemoglobin (HbA1c) for diabetes and the measurement of systolic and diastolic blood pressure for hypertension. Individuals without diabetes were those without self-reported diabetes combined with the non-use of medications and HbA1c < 6.5%; individuals with controlled diabetics were those with self-reported diabetes combined with the use of medications and HbA1c < 7.0%; and individuals with uncontrolled diabetics were those with self-reported diabetes combined with the use of medications and HbA1c ≥ 7.0% [28]. Individuals without hypertension were those without self-reported hypertension combined with the non-use of medication, systolic blood pressure < 140mmHg and diastolic blood pressure < 90mmHg; individuals with controlled hypertension were those with self-reported hypertension combined with the use of medications, systolic blood pressure < 140mmHg and diastolic blood pressure < 90mmHg; individuals with uncontrolled hypertension were those with self-reported hypertension combined with the use of medications, systolic blood pressure ≥ 140mmHg and diastolic blood pressure ≥ 90mmHg [29].

Depressive symptoms were evaluated using the short *Center for Epidemiologic Studies Depression Scale*, with ≥ 4 points considered indicative of the risk of depression [30]. Cognition was evaluated using the word-list learning test, which is divided into two parts: immediate recall (participant hears 10 words and repeats them immediately) and delayed recall (participant is asked to recall the words after three minutes). The score is obtained by the number of words correctly cited on both parts of the test and ranged from 0 to 20 [19, 20].

Waist circumference was measured with a metric tape positioned at the midpoint between the last rib and iliac crest during the expiratory phase with the participant standing, arms alongside the body and trunk unclothed. Waist circumference was considered increased when > 102 cm for men and > 88 cm for women [31]. The body mass index (BMI) was categorized following the classification proposed by the World Health Organization (WHO): underweight (< 18.5 kg/m^2^), normal weight (≥ 18.5 kg/m^2^ and < 25 kg/m^2^), overweight (≥ 25 kg/m^2^ and < 30 kg/m^2^) and obesity (≥ 30 kg/m^2^) [31].

For the collection of the biochemical measures, the participants were asked to fast (except water) for 5 h [32]. Hypercholesterolemia was recorded when total cholesterol was ≥ 190mg/dl [33]. Insulin-like growth factor 1 (IGF-1) was classified following the recommendation by Stenholm and colleagues [34] per age group and sex [32]. Dehydroepiandrosterone sulfate (DHEA-S) was considered low when lower than the 20th percentile of the sample distribution stratified by sex [34].

Season of the year in which blood was collected for the determination of serum 25(OH)D was recorded and categorized as high solar incidence (June to November) and low solar incidence (December to May) in England. Vitamin D supplementation, the use of carbamazepine [anticonvulsant with potential to lower serum 25(OH)D levels] and appendicular skeletal muscle mass index (ASMI) were also used as control variables [35–38]. The ASMI was estimated using Lee’s equation and the cutoff point was the 20th percentile of the sample distribution (< 9.33 kg/m^2^ for men and < 6.66 kg/m^2^ for women) [9, 37–39].

### Statistical Analyses

For the characterization of the sample, continuous variables were expressed as means and standard deviations and categorical variables were expressed as percentages. Differences between 25(OH)D strata among non-dynapenic individuals at baseline were determined using the chi-square test and analysis of variance with Tukey’s post hoc test*.*

For the calculation of incidence densities, the numerator was the number of individuals who developed dynapenia in the period analyzed and the denominator was the sum of the observation time of this population. Poisson regression models were also run because they offer better consistency and efficiency than logistic regression for estimating relative risk in longitudinal studies [40]. The unadjusted model was used to determine the association between level of serum 25(OH)D and the incidence of dynapenia after 4 years of follow-up. The final fully adjusted model was adjusted by seasonality, the use of carbamazepine, vitamin D supplementation, ASMI and incorporated sociodemographic characteristics, behavioral characteristics, clinical conditions and anthropometric and biochemical measures. To test the second hypothesis of the present study, the sensitivity model excluded individuals with osteoporosis and those who were taking vitamin D supplementation. The *Stata 14®* statistical package *(StataCorp*, College Station, TX, USA) was used for the data analysis. The multicollinearity between variables was evaluated by the variance inflation factor (VIF) and considered present when > 10 [41].

## Results

The baseline sample was composed of 3,205 individuals free of dynapenia, 30.0% (95% CI 28.4–31.5) and 22.2% (95% CI 20.7–23.6) of whom had serum 25(OH)D insufficiency and deficiency, respectively. The individuals with serum 25(OH)D deficiency had lower wealth, drank less alcoholic beverages, smoked more, had more depressive symptoms and had a higher mean BMI in comparison to those whose 25(OH)D serum level was categorized as sufficient or insufficient. Moreover, the prevalence of stroke, uncontrolled diabetes and uncontrolled hypertension was higher among individuals with serum 25(OH)D deficiency in comparison to those with sufficient 25(OH)D. No statistically significant difference was found in the prevalence of obesity and increased waist circumference, mean ASMI and collection of the 25(OH)D sample in the period of low solar incidence between individuals with 25(OH)D deficiency and insufficiency, but these figures were significantly higher in both groups compared to individuals with sufficient 25(OH)D. No statistically significant differences were found regarding the daily consumption of alcohol, prevalence of osteoporosis, ideal BMI range and low ASMI between individuals with 25(OH)D deficiency and insufficiency, but these figures were significantly lower in both groups compared to individuals with sufficient 25(OH)D (Tables 1 and 2).

**Table 1:** Sociodemographic characteristics, behavioral factors and health conditions of 3205 participants of ELSA study who were non-dynapenic at baseline stratified by serum 25(OH)D, 2012

	Total *n* = 3205	> 50 nmol/L *n* = 1533 (47.8%)	30–50 nmol/L *n* = 961 (30.0%)	< 30 nmol/L *n* = 711 (22.2%)
*Socioeconomic aspects*				
Age, years (SD)	67.4 ± (7.6)	67.3 ± (7.2)	67.5 ± (7.5)	67.7 ± (8.4)
Sex (female), (%)	53.9	52.9	52.0	58.5
Race (white), (%)	98.3	99.4	98.0^a^	96.1^a^
Not living alone, (%)	94.6	94.2	95.7	94.2
Total wealth, quintiles (%)				
Highest wealth quintile	26.0	29.1	26.3	18.7^a,b^
2nd quintile	23.3	25.2	24.7	17.3^a,b^
3rd quintile	21.6	22.8	20.4	20.7
4th quintile	16.1	14.0	15.2	21.8^a,b^
Lowest wealth quintile	11.2	7.4	11.1^a^	19.5^a,b^
Educational level, (%)				
> 13 years of schooling	34.6	35.1	35.8	31.8
12–13 years of schooling	28.0	27.7	29.0	27.4
0–11 years of schooling	37.4	37.2	35.2	40.8
*Behavioral*				
Physical activity level (active), (%)	97.4	97.7	98.1	96.1
Alcohol intake per week, (%)				
Never or rarely	16.7	13.6	16.0	24.3^a,b^
Often	41.8	42.2	43.2	38.8
Daily	36.0	40.3	34.8^a^	28.5^a^
Smoking, (%)				
Non-smoker	37.8	38.8	38.4	34.7
Ex-smoker	52.4	54.5	51.7	48.8
Smoker	9.8	6.7	9.9^a^	16.5^a,b^
*Health conditions*				
Stroke, yes (%)	2.8	2.1	2.7	4.5^a^
Osteoarthritis, yes (%)	38.2	38.3	36.4	40.5
Cancer, yes (%)	4.8	5.5	3.6	4.5
Diabetes mellitus, yes (%)				
Controlled diabetic	6.7	6.0	4.7	6.9
Uncontrolled diabetic	5.8	5.0	7.6	9.0^a^
Heart disease, yes (%)	15.3	14.9	16.1	14.9
Lung disease, yes (%)	12.7	11.7	12.1	15.8
Arterial hypertension, yes (%)				
Controlled hypertension	28.3	27.9	29.7	27.6
Uncontrolled hypertension	31.4	28.7	32.6	35.8^a^
Osteoporosis, yes (%)	7.9	10.0	5.5^a^	6.5^a^
Depressive symptoms, yes (%)	8.8	6.8	8.5	13.5^a,b^

Data expressed as mean and standard deviation for quantitative variables and percentages for categorical variables

^a^statistically different from 25(OH)D sufficiency (*p* < 0.05)

^b^statistically different from 25(OH)D insufficiency (*p* < 0.05)

**Table 2:** Anthropometric characteristics, biochemical measures and use of medications in 3205 participants of ELSA study who were non-dynapenic at baseline stratified by serum 25(OH)D, 2012

	Total (n = 3205)	> 50 nmol/L 1533 (47.8%)	30–50 nmol/L 961 (30.0%)	< 30 nmol/L 711 (22.2%)
*Anthropometric characteristics*				
Body mass index, kg/m^2^ (SD)	27.9 ± (4.8)	27.2 ± (4.2)	28.2 ± (4.7)^a^	29.1 ± (5.8)^a,b^
Ideal range, (%)	30.0	30.1	24.9^a^	23.1^a^
Underweight, (%)	0.8	1.0	0.5	0.8
Overweight, (%)	43.6	45.6	43.6	39.1^a^
Obesity, (%)	28.6	23.3	31.0^a^	37.0^a^
Waist circumference, (%)				
> 102 cm (men) and > 88 cm (women)	50.4	44.7	54.2^a^	57.8^a^
Men, cm (SD)	101,2 ± (11,4)	99,8 ± (10,4)	101,7 ± (11,2)	103,9 ± (13,5)
Women, cm (SD)	91,1 ± (12,8)	88,9 ± (11,7)	92,2 ± (12,5)	94,1 ± (14,3)
Grip strength, kg (SD)	32.1 ± (10.3)	32.2 ± (10.2)	32.4 ± (10.1)	31.2 ± (10.6)
Men	40.5 ± (8.1)	40.6 ± (7.8)	40.4 ± (7.9)	40.6 ± (8.7)
Women	24.8 ± (5.1)	24.8 ± (4.6)	25.1 ± (5.2)	24.4 ± (5.3)
ASMI, kg/m^2^ (SD)	8.9 ± (1.7)	8.8 ± (1.6)	9.0 ± (1.7)^a^	9.1 ± (1.9)^a^
< 9.33 kg/m^2^ (men) and < 6.66 kg/m^2^ (women)	17.4	20.2	14.9^a^	14.9^a^
*Biochemical measures*				
DHEA-S, low (%)	18.6	16.8	19.3	21.5
Hypercholesterolemia, yes (%)	74.5	74.2	75.3	74.1
IGF-1, low (%)	8.1	7.5	8.0	9.7
*Medications*				
Carbamazepine, yes (%)	2.2	2.0	2.5	2.4
Vitamin D supplementation, yes (%)	4.9	4.9	5.2	4.4
*Seasonality*				
Low solar incidence, (%)	70.0	63.9	73.5^a^	78.6^a^

Data expressed as mean and standard deviation for quantitative variables and percentages for categorical variables

*ASMI* appendicular skeletal muscle mass index, *DHEA-S* dehydroepiandrosterone sulfate (20^th^ percentile of sample distribution), *IGF-1* insulin-like growth factors type 1

^a^statistically different from 25(OH)D sufficiency (*p* < 0.05)

^b^statistically different from 25(OH)D insufficiency (*p* < 0.05)

The incidence density of dynapenia in the 4 years of follow-up was 13.1/1000 individuals/year among those with sufficient 25(OH)D, 20.2/1000 individuals/year among those with insufficiency and 27.4/1000 individuals/year among those with deficiency. The fully adjusted model revealed that 25(OH)D deficiency (< 30 nmol/L) increased the risk of the incidence of dynapenia by 70% (IRR = 1.70; 95% CI 1.04–2.79) in the period analyzed (Table 3—Fully Adjusted Model). Moreover, being 70–79 years of age (IRR = 3.89; 95% CI 1.61–9.44), being 80 years of age or older (IRR = 8.07; 95% CI 3.05–21.36), having osteoporosis (IRR = 1.76; 95% CI 1.02–3.03) and having low IGF-1 (IRR = 1.76; 95% CI 1.02–3.04) were also risk factors for the incidence of dynapenia.

**Table 3:** Adjusted Poisson regression models for incidence of dynapenia over four-year follow-up stratified by 25(OH)D concentrations (2012–2016), ELSA Study

Serum 25(OH)D	Unadjusted model (*n* = 3205) 115 Incident cases	Fully adjusted model (*n* = 3205) 115 Incident cases	Sensitivity model (*n* = 2982) 90 Incident cases
> 50 nmol/L	1.00	1.00	1.00
30–50 nmol/L	1.52 (0.98–2.35)	1.55 (0.99–2.43)	1.77 (1.06–2.94)
< 30 nmol/L	2.01 (1.28–3.16)	1.70 (1.04–2.79)	1.78 (1.01–3.13)

Fully adjusted model: adjusted by seasonality, use of carbamazepine, vitamin D supplementation, ASMI, age, sex, total wealth, living alone, alcohol intake, smoking, physical activity, hypertension, diabetes, osteoporosis, arthritis, stroke, heart disease, lung disease, cancer, depressive symptoms, cognition, waist circumference, DHEA-S, IGF-1 and hypercholesterolemia; Sensitivity Model: excluding individuals with osteoporosis and/or those who performed vitamin D supplementation

When individuals with osteoporosis and those taking vitamin D supplementation were removed from the analyses (*n* = 223), both serum 25(OH)D deficiency (IRR = 1.78; 95% CI 1.01–3.13) and insufficiency (IRR = 1.77; 95% CI 1.06–2.94) were risk factors for the incidence of dynapenia (Table 3—Sensitivity Model). Moreover, being 70–79 years of age (IRR = 5.15; 95% CI 1.77–14.97), being 80 years of age or older (IRR = 8.55; 95% CI 2.66–27.51), having low IGF-1 (IRR = 1.94; 95% CI 1.05–3.61), having low ASMI (IRR = 2.12; 95% CI 1.07–4.19) and having an increased waist circumference (IRR = 1.97; 95% CI 1.08–3.59) were also risk factors for dynapenia.

Finally, the variance inflator factor (VIF) was calculated given that several factors were statistically associated with dynapenia incidence in the fully adjusted models. We found the mean VIF of 1.30 with the variables associated with dynapenia incidence showing VIF values from 1.02 to 2.41. Therefore, the values were within the O'brien´s criteria confirming that there was no collinearity [41].

## Discussion

The main findings of the present study were that 25(OH)D deficiency was a risk factor for the incidence of dynapenia in a 4-year follow-up period. Moreover, when individuals with osteoporosis and those taking vitamin D supplementation were removed from the analyses, both 25(OH)D deficiency and insufficiency were risk factors for the incidence of dynapenia.

There is a paucity of epidemiological evidence that has demonstrated the association between low 25(OH)D concentrations and dynapenia in individuals aged 50 or older. However, such association has been reported in cross-sectional studies, a study design inappropriate to establish causality. Furthermore, these studies used different cut-off points both for the definition of dynapenia and the definition of 25(OH)D deficiency and insufficiency [11–15].

For example, Orces analyzed 2205 Ecuadorians aged 60 or older and found that men and women with grip strength < 26kg and < 16kg, respectively, had a 31% and 43% greater likelihood of having low serum 25(OH)D concentrations (< 50nmol/L) in models controlled by age, race, schooling, BMI, area of residence, smoking habit, consumption of dairy products, level of physical activity and number of diseases [11]. Similarly, Aspell et al. analyzed 4157 participants of the English Longitudinal Study of Ageing between 60 and 90 years of age and found that those with 25(OH)D < 30nmol/L were 44% more likely to low grip strength (< 26kg for men and < 16kg for women) in models controlled by age, sex and physical activity level [12].

Conzade et al. were the only authors to test the association between 25(OH)D deficiency (< 25nmol/L) and low neuromuscular strength (< 30kg for men and < 20kg for women) both cross-sectionally and longitudinally. In the cross-sectional analysis, the authors analyzed 975 participants of the *KORA-Age* study between 65 and 93 years of age and found that 25(OH)D deficiency increased the likelihood of low neuromuscular strength by 59% in models controlled for age, sex, malnutrition risk, physical activity level, BMI and the use of vitamin D supplements. In the longitudinal analysis, 702 participants were followed-up for 3 years. However, 25(OH)D deficiency was not associated with the incidence of dynapenia in models controlled by the same variables considered in the cross-sectional analysis [15].

The fact that we found an association between 25(OH)D deficiency and the incidence of dynapenia while the study cited above found no association in the longitudinal analysis may be explained by differences in the characteristics of the samples, such as age and the prevalence of vitamin D supplementation, as well as methodological issues, such as the cutoff points adopted for the definition of 25(OH)D deficiency and dynapenia [15]. For instance, as the sample in the study by Conzade et al. was older than that of the present investigation (75.7 vs. 67.4 years of age) and the authors used higher cutoff points to define dynapenia (< 30 vs. < 26kg for men and < 20 vs. < 16kg for women), more individuals were excluded at baseline, which should be better for accompanying decline over time. However, the sample in the study by Conzade and colleagues made use of vitamin D supplementation nearly threefold higher than the present sample (13.5 vs. 4.9%) and the cutoff point for defining 25(OH)D deficiency was lower (≤ 25 vs. < 30nmol/L), which may have compromised the longitudinal associations [15].

Furthermore, after excluding participants with osteoporosis and those who were taking vitamin D supplementation, we found that besides deficiency, 25(OH)D insufficiency was also a risk factor for dynapenia, showing that a threshold ≤ 50nmol/L would be sufficient to compromise neuromuscular strength.

Vitamin D participates in both biochemical processes for the maintenance of muscle mass and muscle contraction kinetics. Through its genomic effects, vitamin D exerts an influence on the expression of contractile proteins and differentiation of muscle fibers, which affects the quantity of muscle mass. Through its non-genomic effects, it participates in the regulation and transport of calcium, which exerts an influence on muscle contraction kinetics. Therefore, through two distinct but interlinked mechanisms, vitamin D plays a role in the generation of neuromuscular strength [6, 16, 17]. Hence, its deficiency or insufficiency reduces the uptake of calcium ions (Ca^2+^) in myocytes, interfering with the bonding of these ions to troponin, which compromises contraction and, consequently, the generation of neuromuscular strength [42, 43]. Similarly, 25(OH)D deficiency and insufficiency imply the negative regulation of myogenic markers and transcription factors related to muscle hypertrophy, which hinders muscle repair in the maturation stage as well as the metabolism of mature muscle fibers, inducing and intensifying muscle atrophy, which also contributes to the reduction in neuromuscular strength [44–46].

The present study has strengths and limitations that should be considered. One of the strengths is the fact that this is the first longitudinal study to analyze whether 25(OH)D insufficiency and deficiency are risk factors for the incidence of dynapenia in individuals aged 50 or older. Second, our sample is large and representative of the community-dwelling older English adults. We also included a wide range of sociodemographic, behavioral, clinical and biochemical variables in the adjustment of the statistical models. Finally, in the identification of dynapenia, we considered the handgrip strength cutoff points recommended by the Foundation for the National Institutes of Health Sarcopenia Project (FNIH) that are strongly associated with adverse outcomes closely linked to the musculoskeletal system [23, 26]. In addition, the reference values adopted for classifying the serum concentration of 25(OH)D are also more recommended for the evaluation of musculoskeletal outcomes and meet the needs of 97.5% of the population, while higher values are not consistently associated with benefits [21, 42].

As limitations, the ELSA study only included community-dwelling participants, which limits the external validity of the results, as institutionalized individuals tend to have lower neuromuscular strength, less exposure to sunlight and, consequently, lower serum 25(OH)D concentrations. Although inevitable in longitudinal studies, the losses to follow-up can be a source of bias. Some limitations can also be attributed to ethnicity, activity level and solar incidence of the English community. In this population, white individuals are the majority, which makes comparison and analysis with other ethnicities difficult. The way in which the level of physical activity was classified (active vs. inactive) can also influence the analyses. However, it has already been used in other studies and was obtained through the Health Survey for England, a validated instrument that is widely used in research [26]. We also found a higher proportion of 25(OH)D collection in periods of low solar incidence among 25(OH)D insufficient and deficient individuals, and this proportion was statistically higher and different from individuals with 25(OH)D sufficiency. Aware that low sunlight compromises the synthesis of 25(OH)D, it is possible that the prevalence of 25(OH)D insufficiency and deficiency in our sample is overestimated and, consequently, this may bring some degree of bias in our results.

Finally, the ELSA study does not include two important control variables: parathormone (PTH) and creatinine. PTH is high in individuals with 25(OH)D deficiency, characterizing secondary hyperparathyroidism, which is associated with the reduction in neuromuscular strength [3]. High creatinine indicates kidney failure, which can interfere in the metabolism of 25(OH)D, contributing to a reduction in its levels. These variables could be included in future analysis. Despite this, the association between 25(OH)D deficiency and the incidence of dynapenia was significant.

So we concluded that older adults with serum 25(OH)D levels < 30 nmol/L are at greater risk of the incidence of dynapenia than those with serum levels ≥ 30 nmol/L. However, when we excluded individuals with osteoporosis and those who were taking vitamin D supplementation, the risk threshold for the incidence of dynapenia was higher (≤ 50 nmol/L). Clinical trials that combine resistance exercises and vitamin D supplementation in individuals with dynapenia and 25(OH)D insufficiency or deficiency should be developed to investigate whether this combined therapy increases muscle strength in individuals aged 50 or older.
